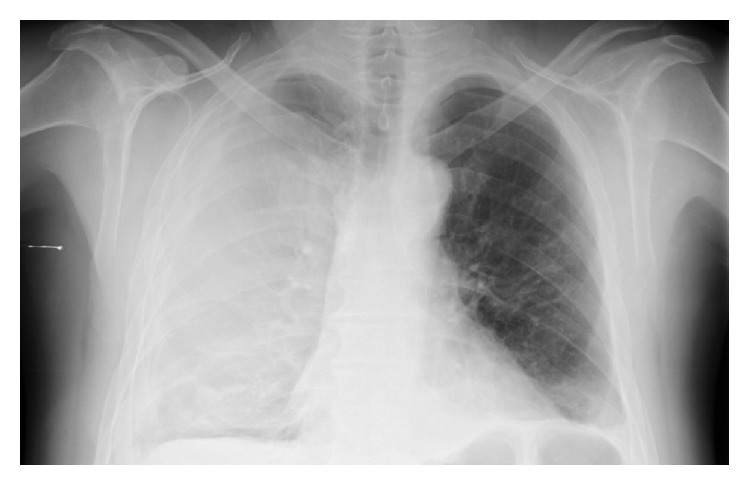# Massive Bleeding as the First Clinical Manifestation of Metastatic Prostate Cancer due to Disseminated Intravascular Coagulation with Enhanced Fibrinolysis

**DOI:** 10.1155/2016/7217915

**Published:** 2016-10-10

**Authors:** Mónica Palma Anselmo, Gustavo Nobre de Jesus, João Madeira Lopes, Rui M. M. Victorino, João Meneses Santos

**Affiliations:** ^1^Internal Medicine Department 2, Hospital de Santa Maria, Lisboa, Portugal; ^2^Faculdade de Medicina de Lisboa, Lisboa, Portugal

## Abstract

Disseminated intravascular coagulation (DIC) is the most frequent coagulation disorder associated with metastatic prostate adenocarcinoma. However, DIC with enhanced fibrinolysis as an initial presentation of prostate cancer is extremely rare. The appropriate treatment to control bleeding in these situations is challenging, controversial, and based on isolated case reports in the literature. A 66-year-old male presented at the emergency department with acute severe spontaneous ecchymoses localized to the limbs, laterocervical hematoma, and hemothorax. Prostate specific antigen level was 385 *μ*g/L, bone scintigraphy revealed multiple bone metastases, and prostate biopsy confirmed adenocarcinoma (Gleason 9; 4 + 5). Laboratory investigation showed a pattern of enhanced fibrinolysis rather than the more common intravascular coagulation mechanism. Epsilon aminocaproic acid in monotherapy was initiated with a clear and rapid control of bleeding manifestations. This rare case of massive bleeding due to DIC with enhanced fibrinolysis as the first manifestation of prostate cancer suggests that in selected cases where the acute bleeding dyscrasia is clearly associated with a dominant fibrinolysis mechanism it is possible to use an approach of monotherapy with antifibrinolytics.

## 1. Introduction

Disseminated intravascular coagulation (DIC), as defined by The Scientific Standards Committee of the International Society on Thrombosis and Hemostasis [1], is “an acquired syndrome characterized by the intravascular activation of coagulation with loss of localization arising from different causes.” Physiopathology of the disease lays on an abnormal activation of coagulation with consumption of coagulation factors and platelets and a process of hyperfibrinolysis. The degree of fibrinolytic activation differs in the DIC type, namely, 3 types: DIC with suppressed fibrinolysis, DIC with enhanced fibrinolysis, and an intermediate pathogenesis between the last two—the balanced fibrinolysis type [2]. DIC with enhanced fibrinolysis is the less frequent type and there is evidence of a link between prostate cancer and this subtype of DIC [3]. We present a case of a disseminated prostate cancer where the initial manifestation was uncontrolled hemorrhage secondary to DIC with enhanced fibrinolysis, successfully treated with epsilon aminocaproic acid without heparin.

## 2. Case Presentation

A 66-year-old white male was admitted with spontaneous ecchymoses over his limbs and a 10 cm hematoma on left laterocervical and supraclavicular region. Rectal examination revealed a large and hard prostate. No clinical signs of infection or hepatic failure were found. Past medical history included arterial hypertension and benign prostatic hyperplasia, and there was neither antiplatelet nor anticoagulant therapy. Two months earlier, prostate specific antigen (PSA) level was 40 *μ*g/L, without identifiable malignancy on prostate biopsy, collected from six different locations.

Laboratorial investigation showed hemoglobin 12.1 g/dL, leukocytes 9340/*μ*L, platelets 115000/*μ*L, prothrombin time 17.1/11.6 seconds, activated partial thromboplastin time 33.4/29.0 seconds, D-dimer 61.77 *μ*g/mL, and fibrinogen 72 mg/dL. PSA level was 385 *μ*g/L. Creatinine, urea, and liver enzymes were normal.

Transrectal echography was performed revealing a heterogenous prostate with discrete exophytic component. Prostate biopsy identified adenocarcinoma (Gleason 9: 4 + 5). A thoracoabdominopelvic tomography scan revealed no visceral metastization and bone scintigraphy confirmed the presence of lytic and blastic lesions of left scapula, body of L4 and L5 and sacral region.

The diagnosis of DIC with enhanced fibrinolysis was made and the patient started bicalutamide and subsequently received leuprolide. Fibrinogen was administered if below 100 mg/dL. After 5 days of treatment there was a remission of hemorrhagic dyscrasia, coagulation parameters improved, and the patient was discharged home.

He was readmitted two days later with a massive right hemothorax (Figure 1). Hemoglobin level was 4.8 g/dL, hematocrit 14.0%, leukocyte count 13000/*μ*L, platelet count 154000/*μ*L, prothrombin time 15.1/11.6 seconds, activated partial thromboplastin time 21.8/29 seconds, D-dimer 37.42 *μ*g/mL, and fibrinogen 100 mg/dL. A chest tube was inserted and supportive treatment was initiated with packed red blood cells, fresh-frozen plasma, and fibrinogen transfusions over 3 days; however, drainage remained active. IV epsilon aminocaproic acid (EACA) was then started (2.5 g* t.i.d.*) with progressive reduction of the chest tube drainage. After 48 hours of treatment with EACA, the chest tube was removed and the patient was discharged with a hemoglobin level of 8.0 g/dL, platelet count of 247000/*μ*L, prothrombin time 15.1/11.6 seconds, fibrinogen level of 276 mg/dL, and D-dimer of 7.8 *μ*g/mL. Patient had no recurrence of bleeding over the 5 months of follow-up with hormonotherapy with bicalutamide and leuprolide. Six months after the diagnosis, as a result of brain trauma, the patient died of intracranial hemorrhage.

## 3. Discussion

DIC results from an imbalance between coagulation and fibrinolysis and it is currently classified in subtypes according to primary coagulopathy disorder [2, 4–6]. Thus, DIC can be divided into three subtypes: DIC with suppressed fibrinolysis, in which coagulation activation is severe but fibrinolysis is mild, a predominantly procoagulant subtype seen, for instance, in pancreatic cancer; DIC with balanced fibrinolysis, often subclinical with laboratory abnormalities, well described in context of many solid tumors; and DIC with enhanced fibrinolysis, where bleeding is the characteristic clinical manifestation. This latter form, where hyperfibrinolysis is predominant, is characterized by more elevated D-dimer, absence of severe platelet depletion, and fibrinogen level below 100 mg/dL. It is well recognized in acute promyelocytic leukemia or, as in our case, metastatic prostate cancer [2].

DIC is the most frequent coagulopathy disorder in patients with prostate cancer, described in 13 to 30%, but is often subclinical and only 0.4–1.65% of these have a clinical expression [7–9]. Moreover, DIC as first manifestation of prostate cancer is extremely rare [8, 9]. The incidence depends on the tumor stage, is enhanced in metastatic hormone-refractory disease [7, 8], and occurs more frequently following prostate manipulation [10, 11]. The precise mechanisms of DIC in prostate cancer are not fully clear but tissue factors and urokinase-type plasminogen activator produced by the tumor are thought to have an important role in coagulation and fibrinolytic pathways [12]. The subtype DIC with enhanced fibrinolysis, described in metastatic prostate cancer, is associated with strong fibrinolytic activation as response to coagulation activation, which explains the severe bleeding symptoms [13–15]. Within this subtype, the vector for hyperfibrinolysis is clearly predominant in the axis of coagulopathy. Our patient had a rare presentation of a metastatic prostate cancer with this DIC subtype, since there was mainly enhanced fibrinolysis, as shown by prolonged plasma coagulation time, mild platelet depletion, and low fibrinogen.

Understanding the mechanism of the coagulopathy is the key to choose therapeutic management on specific cases. Treatment remains controversial as guidelines are not unequivocal [16–18] and studies are contradictory [19]. The therapeutic cornerstone of this disturbance of coagulation is the treatment of the primary illness. Although castration therapy is mandatory in hormone sensitive patients, sometimes it is not sufficient to stop life-threatening episodes of bleeding. In a series of 43 DIC patients with prostate cancer, the majority had metastatic disease and was resistant to castration [20]. The median survival time in patients treated with a combination of heparin and chemotherapy was 4 weeks, less than our patient. Hyman et al. reported that more than half of prostate cancer patients who developed DIC had high grade disease. In fact, it has been postulated that increased fibrinolysis promotes metastasis [9] and therefore relates with poor prognosis [21]. Both statements are consistent with what we found in our patient: a Gleason score 9 with bone metastasis and a predominant fibrinolytic pattern. Although evidence is limited, patients with DIC with enhanced fibrinolysis may benefit from antifibrinolytic agents, especially if massive bleeding persists.

It is known that trauma, particularly closed head injury, is associated with DIC [14]. Hyperfibrinolysis plays an important role at an early stage of trauma and contributes to severe bleeding and consequently poor prognosis [22]. Our patient had a latent/subclinical state of DIC that could have been potentiated by the trauma insult.

In conclusion, we present a case that illustrates an atypical initial presentation of prostate carcinoma consisting of massive bleeding due to DIC with excessive fibrinolysis that was successfully treated with EACA in monotherapy without further supportive therapy and with neither thrombotic nor spontaneous bleeding phenomena. We suggest that in cases of DIC with acute presentation as massive bleeding where there is good evidence that the predominant mechanism is enhanced fibrinolysis an attempt monotherapy with EACA may be justified.

## Figures and Tables

**Figure 1:**